# Update of the species checklist of *Culicoides* Latreille, 1809 biting midges (Diptera: Ceratopogonidae) of Morocco

**DOI:** 10.1186/s13071-019-3720-4

**Published:** 2019-09-24

**Authors:** Maria Bourquia, Claire Garros, Ignace Rakotoarivony, Laetitia Gardès, Karine Huber, Intissar Boukhari, Jean-Claude Delécolle, Thierry Baldet, Antoine Mignotte, Youssef Lhor, Khalid Khallaayoune, Thomas Balenghien

**Affiliations:** 1 Institut Agronomique et Vétérinaire Hassan II, Unité Parasitologie et Maladies Parasitaires, 10100 Rabat, Morocco; 2 ASTRE, Univ Montpellier, Cirad, INRA, Montpellier, France; 3 Cirad, UMR ASTRE, 34398 Montpellier, France; 4 Cirad, UMR ASTRE, 97490 Ste Clotilde, La Réunion France; 5 Cirad, UMR ASTRE, 97170 Petit-Bourg, Guadeloupe France; 6 Institut de Parasitologie et de Pathologie Tropicale de Strasbourg (IPPTS) EA7292, 3 rue Koeberlé, 67000 Strasbourg, France; 7 Office National de Sécurité Sanitaire des Produits Alimentaires, Institut Agronomique et Vétérinaire, 10015 Rabat, Morocco; 8 Institut Agronomique et Vétérinaire Hassan II, Unité Microbiologie, Immunologie et Maladies Contagieuses, 10100 Rabat, Morocco

**Keywords:** Species diversity, Inventory, *Culicoides*, Biting midges, Rabat region, Morocco

## Abstract

**Background:**

Investigations of *Culicoides* fauna, including inventories, were carried out in Morocco at different periods after the country had faced major bluetongue and African horse sickness outbreaks. However, no comprehensive reference publication has provided a clear overview of the *Culicoides* species diversity. This study reviewed available data on *Culicoides* biting midge species in Morocco from 1968 to 2015 (published and grey literature in French and English) in order to revise the current checklist, in light of state of the art taxonomic and systematic knowledge, and confirmed the checklist with morphological and molecular identifications of specimens collected from the region of Rabat.

**Methods:**

Literature related to *Culicoides* collections in Morocco was collated. Authors were contacted to obtain raw data and additional information for the collections. Fresh *Culicoides* material was collected and examined from two sites around Rabat, the capital of Morocco. Each collected individual was examined and morphologically identified, if possible, to the species level. In addition, molecular identification was performed to separate closely related species, to confirm difficult morphological identifications and to confirm new species records.

**Results:**

A total of 6121 individuals of *Culicoides* spp. were collected and analyzed and at least 17 species were identified: *C. cataneii/C. gejgelensis*, *C. circumscriptus*, *C. fagineus*, *C. festivipennis*, *C. imicola*, *C. jumineri*, *C. kingi*, *C. longipennis*, *C. montanus*, *C. newsteadi*, *C. obsoletus*, *C. paolae*, *C. parotti*, *C. puncticollis*, *C. sahariensis*, *C. scoticus* and *C. subfagineus*. Seven species were confirmed using phylogenetic analyses. Two new species records for Morocco are reported: *C. paolae* and *C. subfagineus*.

**Conclusions:**

The Moroccan fauna of *Culicoides* now includes 54 valid species. Further work would certainly increase this total, as one of the clades we identified was not affiliated to any described and valid species.

## Background

Biting midges of the genus *Culicoides* Latreille, 1809 (Diptera: Ceratopogonidae) are responsible for the transmission of economically important viruses in animal production, most notably African horse sickness virus (AHSV), bluetongue virus (BTV), epizootic hemorrhagic disease virus (EHDV) and the Schmallenberg virus which emerged in 2011 in Europe [1–4]. Over the past two decades, multiple incursions of BTV serotypes [5] into the Mediterranean basin and the rest of Europe have dramatically changed the epidemiology of *Culicoides*-borne diseases in this region and increased the need for up to date taxonomic knowledge. Indeed, although *Culicoides imicola* Kieffer, 1913 is known as a proven BTV vector species for a very long time [6, 7], recent BTV outbreaks, especially in regions where *C. imicola* is absent, have highlighted that other species are able to transmit BTV. Within species present in the Mediterranean region, some other species of the subgenus *Avaritia*, such as *Culicoides obsoletus* (Meigen, 1818) and *Culicoides scoticus* Downes & Kettle, 1952, are considered probable BTV vectors based on their ecological habits, on virus isolation or viral genome detections from field-collected individuals and on experimental infections [8–10]. Moreover, several species of the subgenus *Culicoides*, such as *Culicoides pulicaris* (Linnaeus, 1758), *Culicoides lupicaris* Downes & Kettle, 1952 *Culicoides punctatus* (Meigen, 1804) or *Culicoides newsteadi* Austen, 1921, are considered possible BTV vectors based on their ecological habits and on virus isolation or viral genome detections from field-collected individuals [11–14]. Finally, BTV genome has been found in the abdomen and thorax of *Culicoides paolae* Boorman, 1996 [15], questioning its role as possible vector.

Previous investigations of the *Culicoides* fauna of Morocco have been prompted by major epizootics of *Culicoides*-borne diseases. The first intensive faunistic inventory started after the large AHSV outbreaks affecting northern Africa and Spain in 1965–66 [16]. Various surveys conducted in 1968–69 and 1970–71 throughout the country reported the existence of many *Culicoides* species and described two new species: *Culicoides calloti* Kremer, Delécolle, Bailly-Choumara & Chaker, 1979 and *Culicoides landauae* Kremer, Rebholtz-Hirtzel & Bailly-Choumara, 1975 [17–21]. Two additional species, *Culicoides clastrieri* Callot, Kremer & Deduit, 1962 and *Culicoides duddingstoni* Kettle & Lawson, 1955, were collected in Fès-Meknès Region in 1972, but the information was not published (Delécolle, pers. comm.). Taking into account the current classification of the genus *Culicoides*, the checklist in the 1970s consisted of at least 54 species (Table 1).

**Table 1:** *Culicoides* species list for Morocco

Species	Reference	Our data	Proposed checklist
*C. azerbajdzhanicus* Dzhafarov, 1962	[3, 4, 6]		✓
*C. badooshensis* Khalaf, 1961	[3, 4, 6]		✓
*C. calloti* Kremer, Delécolle, Bailly-Choumara & Chaker, 1979	[6, 7]		✓
*C. cataneii* Clastrier, 1957	[4, 6]	✓	✓
*C. circumscriptus* Kieffer, 1918	[1, 3, 4, 6, 9]	✓	✓
*C. clastrieri* Callot, 1962	^a^		✓
*C. derisor* Callot & Kremer, 1965	[4, 6]		✓
*C. duddingstoni* Kettle & Lawson, 1955	^a^		✓
*C. dzhafarovi* Remm, 1967	[4, 6]		✓
*C. faghihi* Navai, 1971	[5]		✓
*C. fagineus* Edwards, 1939	[4, 6]	✓	✓
*C. festivipennis* Kieffer, 1914	[1, 3, 4, 6, 9] (as synonym name *C. odibilis*)	✓	✓
*C. helveticus* Callot, Kremer & Deduit, 1962	[10]		
*C. heteroclitus* Kremer & Callot, 1965	[5]		✓
*C. imicola* Kieffer, 1913	[3, 4, 6, 8, 9] (as synonym name *C. pallidipennis*)	✓	✓
*C. jumineri* Callot & Kremer, 1969	[4, 6, 9] (as Jumineri group)	✓	✓
*C. kibunensis* Tokunaga, 1937	[5] (as synonym name *C. cubitalis*)		✓
*C. kingi* Austen, 1912	[2] (as Schultzei/Kingi group)	✓	✓
*C. kurensis* Dzhafarov, 1960	[9]		✓
*C. landauae* Kremer, 1975	[5]		✓
*C. langeroni* Kieffer, 1921	[3, 4, 6]		✓
*C. longipennis* Khalaf, 1957	[4]	✓	✓
*C. marcleti* Callot, Kremer & Basset, 1968	[4]		✓
*C. maritimus* Kieffer, 1924	[3, 4, 6]		✓
*C. montanus* Shakirzjanova, 1962	[4, 6]	✓	✓
*C. newsteadi* Austen, 1921	[1, 3, 4, 6, 9] (as synonym name *C. halophilus*)	✓	✓
*C. obsoletus* (Meigen, 1818)	[1, 3, 4, 6, 9]	✓	✓
*C. odiatus* Austen, 1921	[3, 4, 6–9] (as synonym names *C. lailae* and *C. indistinctus*)		✓
*C. pallidicornis* Kieffer, 1919	[4] (as Achrayi/pallidicornis group)		✓
*C. pallidus* Khalaf, 1957	[4] (as synonym name *C. stackelbergi*)		✓
*C. paolae* Boorman, 1996		✓	✓
*C. parroti* Kieffer, 1922	[3, 4, 6]	✓	✓
*C. pictipennis* (Staeger, 1839)	[3]		✓
*C. picturatus* Kremer & Deduit, 1961	[3, 4, 6]		✓
*C. pseudopallidus* Khalaf, 1961	[3, 4]		✓
*C. pulicaris* (Linnaeus, 1758)	[4, 6, 8, 9]		✓
*C. pumilus* (Winnertz, 1852)	[5]		✓
*C. punctatus* (Meigen, 1804)	[1, 3, 4, 6]		✓
*C. puncticollis* (Becker, 1903)	[1, 3, 4, 6, 9]	✓	✓
*C. ravus* de Meillon, 1936	[4, 6] (as synonym name *C. subravus*)		✓
*C. saevus* Kieffer, 1922	[1, 3, 4, 6]		✓
*C. sahariensis* Kieffer, 1923	[3, 4, 6–9] (as synonym name *C. coluzzi*)	✓	✓
*C. santonicus* Callot, Kremer, Rault & Bach, 1966	[3, 4, 6]		✓
*C. schultzei* (Enderlein, 1908)	[1]		✓
*C. scoticus* Downes & Kettle, 1952	[4, 6]	✓	✓
*C. sejfadinei* Dzhafarov, 1958	[4, 6]		✓
*C. semimaculatus* Clastrier, 1958	[5]		✓
*C. sergenti* Kieffer, 1921	[6] (as synonym name *C. mosulensis*)		✓
*C. shaklawensis* Khalaf, 1957	[5]		✓
*C. similis* Carter, Ingram & Macfie, 1920	[4]		✓
*C. simulator* Edwards, 1939	[5]		✓
*C. subfagineus* Delécolle & Ortega, 1998		✓	✓
*C. subfasciipennis* Kieffer, 1919	[4, 6, 9]		✓
*C. truncorum* Edwards, 1939	[4] (as synonym name *C. sylvarum*)		✓
*C. univittatus* Vimmer, 1932	[3, 4, 6] (as synonym name *C. agathensis)*		✓
*C. vidourlensis* Callot, Kremer, Molet & Bach, 1968	[3]		✓

*Notes*: [1] Callot et al. (1968); [2] Kremer et al. (1970); [3] Kremer et al. (1971); [4] Kremer et al. (1975); [5] Chaker et al. (1979); [6] Kremer et al. (1979); [7] Baylis et al. (1997); [8] Bouayoune et al. (1998); [9] Cêtre-Sossah & Baldet (2004); [10] Lhor et al. (2015)

^a^Unpublished records of Delécolle

**Fig. 1:**
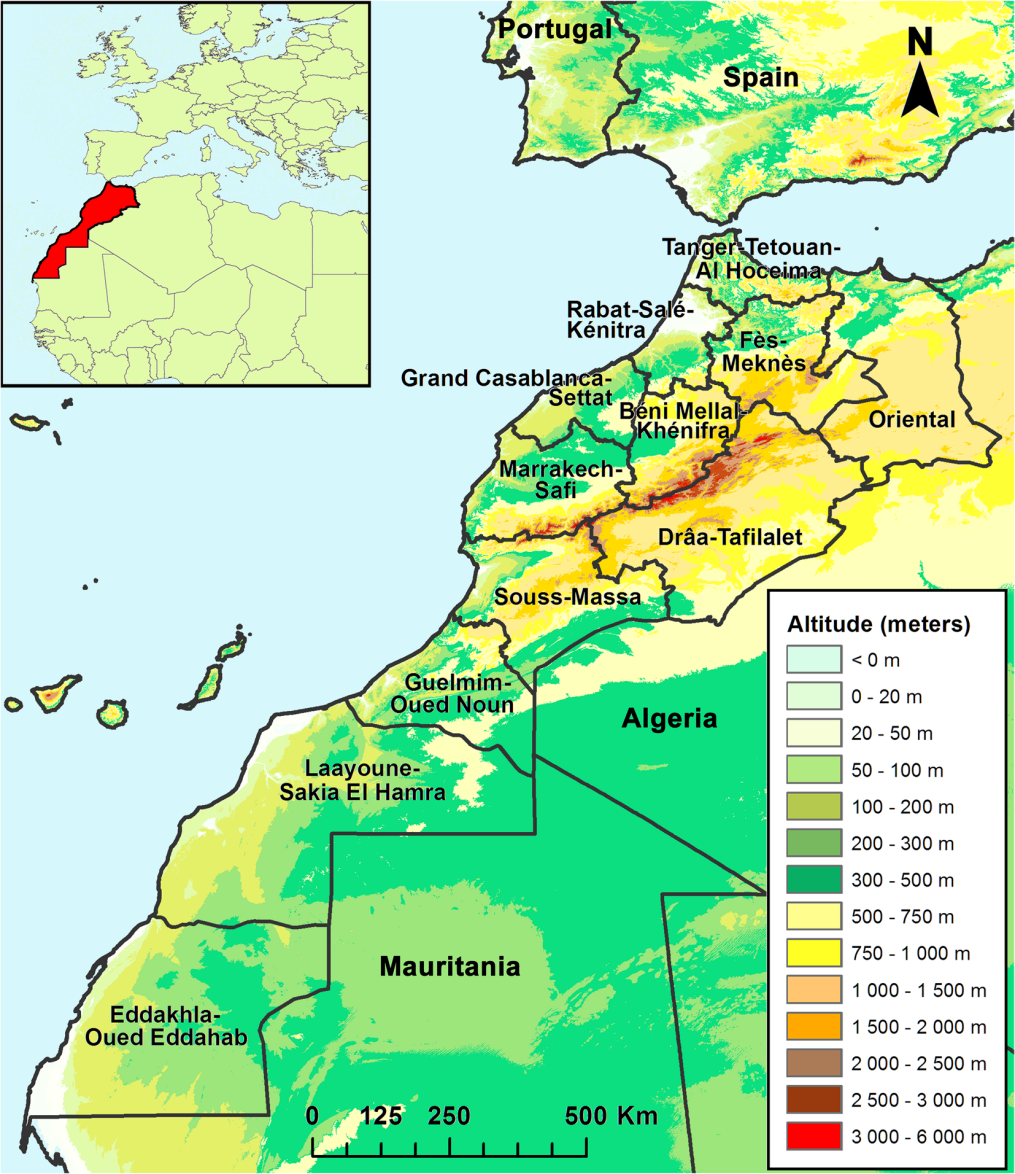
Map of the regions in the Kingdom of Morocco

A second AHS epizootic occurred in Spain in 1987, from where it spread to Morocco in 1989 and persisted until 1991 [22]. At that time, the Afrotropical species *C. imicola* was the only proven AHSV vector and was considered the only species involved in AHSV transmission in Morocco [23, 24]. As a result, surveys conducted in Morocco in 1994–95 were focused on characterizing the distribution and abundance of *C. imicola* [23, 25]. *Culicoides imicola* is very widely dispersed, found at altitudes of up to 1275 meters and under climatic conditions ranging from Mediterranean to Saharan, with the highest catches in the lower areas of the northwest (between Tangier and Rabat) and in Marrakech (Fig. 1). Other species reported were *Culicoides circumscriptus* Kieffer, 1918, *C. newsteadi*, *Culicoides puncticollis* (Becker, 1903) and specimens close to *Culicoides festivipennis* Kieffer, 1914 (mentioned under its synonym name, *Culicoides odibilis* Austen, 1921), but also *C. obsoletus* and *C. pulicaris*. The two latter referred more likely to ‘group’ than to single species. Indeed, authors confirmed that morphologically close species might have been grouped with *C. obsoletus* or *C. pulicaris*, as identification focused mainly on *C. imicola* [23].

The late 1990s were marked by the occurrence of massive and regular BTV outbreaks in the Mediterranean [5]. The scale of these successive epizootic diseases has led to the establishment of entomological surveillance networks dedicated to *Culicoides* in most Mediterranean countries. In Morocco, a cross-sectional study was therefore carried out in 2000–2003 by the veterinary services in 50 sites. This third study confirmed the wide distribution of *C. imicola*, which was the most frequent species in the collections [26], but did not provide additional inventory information, except for *C. imicola*; the samples collected by these authors were identified only at the level of species groups.

The first BTV outbreak in Morocco was reported in September 2004 [27]. Since then, BTV outbreaks have been reported every year, except for 2008, in the entire Moroccan territory, whereas the country faced EHDV outbreaks in 2006. Following snapshot entomological surveys carried out in BTV transmission sites [28], a new entomological surveillance network was set up in 2009–2010 as part of the epidemiological surveillance of animal diseases organized by the national veterinary services [29]. This fourth study resulted in the publication of an updated *Culicoides* checklist for Morocco, with the addition of a new species based on a single morphologically identified specimen: *Culicoides helveticus* Callot, Kremer & Deduit, 1962. The lack of molecular confirmation for this individual alone suggests caution in including this new species in the national checklist.

In the present study, we aimed to revise the current inventory of *Culicoides* species present in Morocco in light of the most up to date taxonomic and systematic knowledge. The studies in the 1970s used species names that are now considered as synonymous. Morphological identification of *Culicoides* is also difficult; variations in diagnostic characteristics are common and could lead to false affiliation of specimens [30, 31]. Therefore, we aimed to confirm the checklist for Morocco based on the examination of fresh material collected in the Rabat region, the Moroccan capital, by an integrative taxonomy approach using morphological identification supplemented by molecular identification to confirm species records.

## Methods

Literature related to *Culicoides* collections in Morocco, including articles published in French, English and unpublished ‘grey’ literature (mainly reports or theses), was collated. Authors were contacted to obtain raw data on the collections and details on the level of identification (species or ‘group’).

In these previous studies, only morphological identification was used. We thus decided to collect and examine fresh *Culicoides* material from two sites around Rabat, a horse-riding center (33°56′N, 6°49′W) (site M1) and a goat farm (33°51′N, 6°51′W) (site M2) to make samples available for molecular identification. The objective was to confirm the record of some species for which morphological identification is difficult. Collection was made using an Onderstepoort Veterinary Institute black light suction trap operated from dusk until dawn, on two consecutive days every two weeks (from May to October 2016). When possible, collected individuals were morphologically identified to the species level using relevant morphological identification keys [32, 33].

Additionally, molecular identification was performed (i) to identify closely related species (such as *C. obsoletus*, *C. scoticus* or *Culicoides montanus* Shakirzjanova, 1962); (ii) to confirm difficult morphological identifications (damaged specimen or uncertain diagnostic features); and (iii) to confirm new species records. Genomic DNA was individually extracted using the NucleoSpin® Tissue DNA Kit (Macherey-Nagel, Duren, Germany) according to the manufacturer’s instructions. For *C. obsoletus/C. scoticus*, the allele-specific PCR from Nolan et al. [34] was used to separate the two morphologically similar species. For other species, *cox*1 amplification and sequencing using primers were produced following procedure described by Bakhoum et al. [35], before final species assignment was done by phylogenetic reconstructions and BLAST search of GenBank entries and an Afrotropical BOLD database (AFCUL001-18 to AFCUL1131-18).

The newly generated *cox*1 sequences were edited in GENEIOUS R6 (Biomatters, http://www.geneious.com/) then aligned using the MUSCLE algorithm [36]. Maximum Likelihood (ML) phylogenetic trees were computed under a substitution model estimated using the JMODELTEST tool within MEGA X [37]. The Bayesian information criterion implemented within JMODELTEST was used to determine the most suitable evolutionary model; this was the general time reversible (GTR) model with gamma distributed (+G) rate variation among sites (5 categories; parameter = 0.6015) and proportion of invariable sites (+I) of 22.82%. Missing data, including indels, were excluded from the analysis. The tree was subjected to 1000 bootstrap replications to assess topological reliability. Initial tree(s) for the heuristic search were obtained automatically by applying Neighbor-Join and BioNJ algorithms to a matrix of pairwise distances estimated using the Maximum Composite Likelihood (MCL) approach, and then selecting the topology with superior log likelihood value.

Specimens were stored in alcohol with one specimen per species slide-mounted and are available upon request to Maria Bourquia and Khalid Khallaayoune at Agronomic and Veterinary Institute Hassan II, Rabat, Morocco. Sequences were deposited in the GenBank database under the accession numbers MK732283–MK732313. Sequences from the GenBank and BOLD databases were used in the tree reconstruction. Sequences for *Culicoides halophilus* Kieffer, 1924 (GenBank: KX853271 and KX853272) were used although not considered as a valid species by the world catalogue of *Culicoides* [38].

## Results

**Fig. 2:**
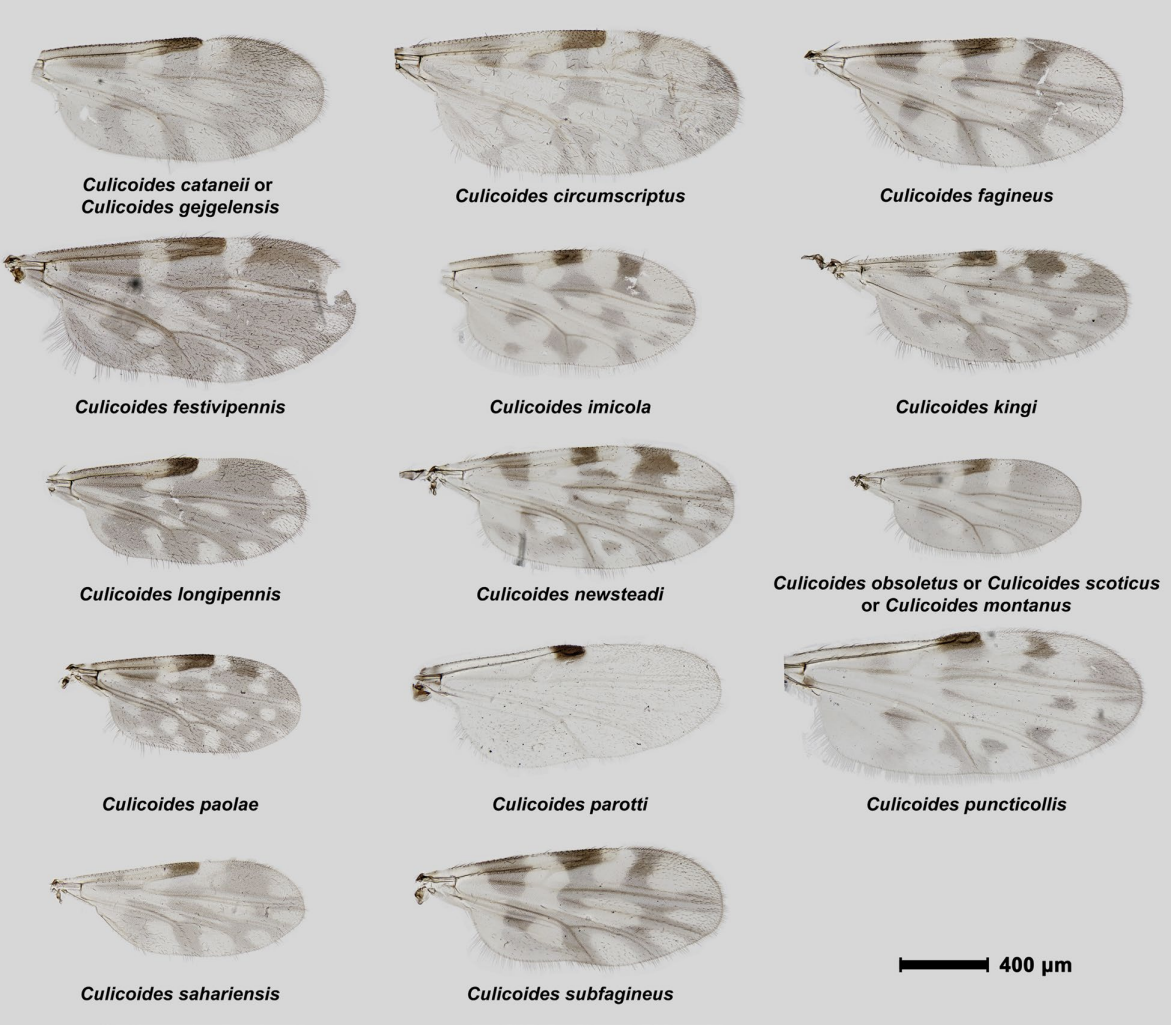
Wing patterns of *Culicoides* spp. collected during the present survey

A total of 6121 *Culicoides* were examined, representing at least 17 species collected during a total of 12 collection sessions. Morphological identification confirmed the presence of 15 previously reported species and established two new records: *C. paolae* with 6 individuals and *Culicoides subfagineus* Delécolle & Ortega, 1998 with 3 individuals (Table 1). We dissected and mounted the wings of the different species including *C. paolae* and *C. subfagineus* (Fig. 2).

Molecular identification was performed on 105 individuals. A total of 71 females morphologically identified as *C. obsoletus*/*C. scoticus* were identified with the species-specific PCR [34] allowing the identification of 59 *C. obsoletus* and 7 *C. scoticus*. Five individuals could not be identified to the species level and were kept as *C. obsoletus*/*C. scoticus*. *cox*1 sequences were generated from 34 individuals of which 3 individuals did not produce exploitable sequences. Alignment was 390 bp long and included 31 sequences plus 135 references sequences retrieved from GenBank, BOLD or expert molecular library.

**Fig. 3:**
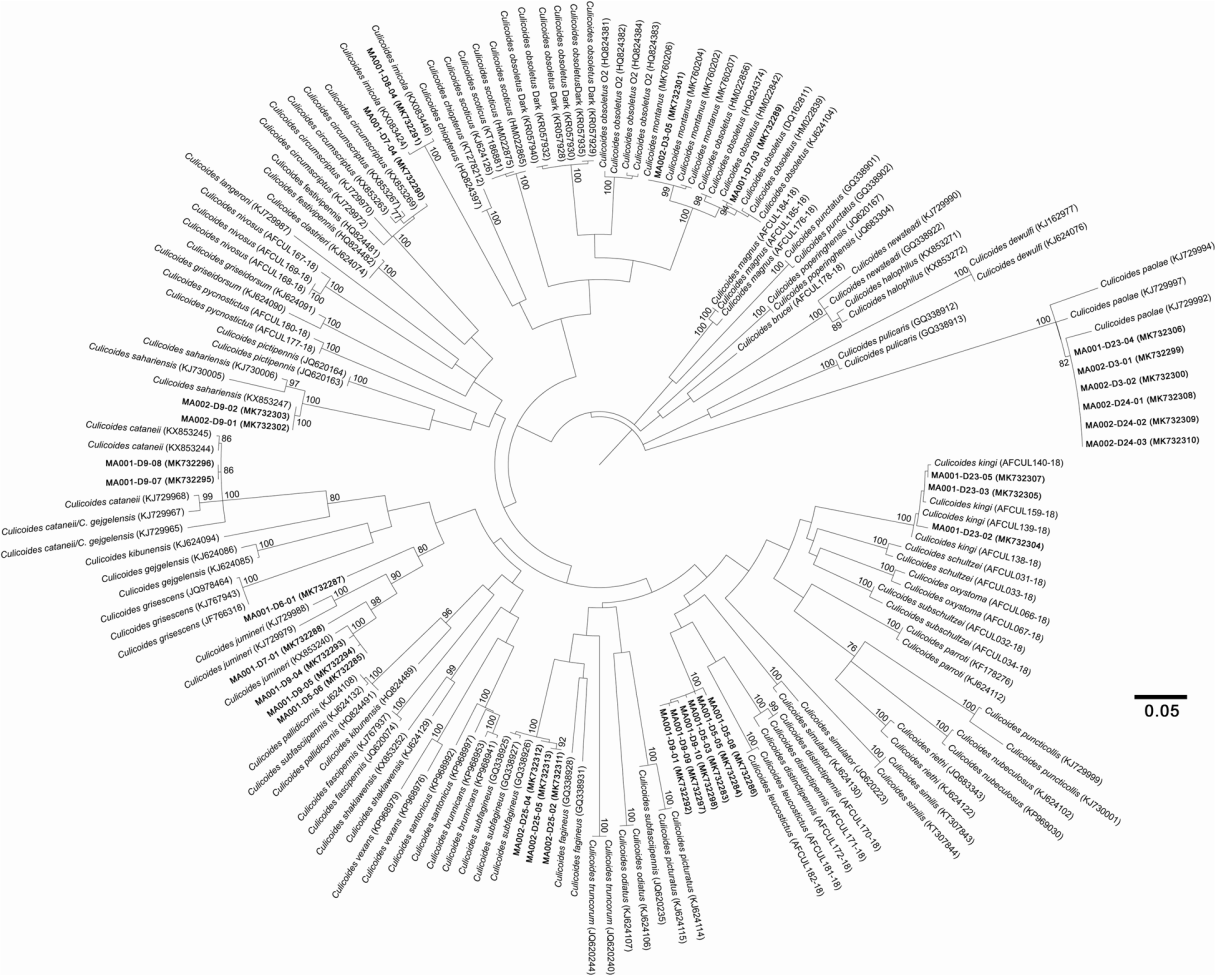
Phylogenetic tree based on *cox*1 polymorphism. In bold, sequences produced in this study with GenBank reference numbers in brackets. The evolutionary history was inferred using the Maximum Likelihood method and GTR+G+I model. This analysis involved 166 nucleotide sequences. All positions containing gaps and missing data were eliminated (complete deletion option); there were a total of 390 positions in the final dataset. The tree is drawn to scale, with branch lengths corresponding to the number of substitutions per site

The resulting tree was used as a species affiliation tool and not to infer systematic relationships. Indeed, the node robustness was high (above 90%) only at the terminal nodes which allows affiliation at the species level. From the tree we infer the presence of *Culicoides cataneii* Clastrier, 1957 (2 sequences), *C. circumscriptus* (1 sequence), *C. imicola* (1 sequence), *Culicoides jumineri* Callot & Kremer, 1969 (5 sequences), *C. montanus* (1 sequence), *C. obsoletus* (1 sequence), *C. paolae* (6 sequences), *Culicoides sahariensis* Kieffer, 1923 (2 sequences), *Culicoides kingi* Austen, 1912 (3 sequences), *C. subfagineus* (3 sequences) and 6 sequences clustering in the same clade without voucher sequence (Fig. 3). Molecular affiliation of two sequences (MK732287 and MK732288) should be considered with caution. They clustered in proximity to *C. jumineri* sequences. However, these two individuals could not be considered with certainty as *C. jumineri*, due to the bootstrap values being too low, highlighting genetic differences between these two sequences and the *C. jumineri* reference sequences.

## Discussion

Species inventories allow completing and updating taxonomic and systematic revision to develop identification keys, establish national or regional species checklists and field guides, and make all the relevant information readily available through scientific publications and other dissemination tools. Regular updates of faunistic inventories of economically important biological groups are of great importance to maintain expertise and overcome taxonomic obstacles, train young scientists and ensure the stability of species community structure in an environment subject to the introduction and colonization of exotic species [39].

The present paper revised, updated and confirmed the checklist of *Culicoides* for Morocco using combined morphological and molecular identification and, to the authors’ knowledge, reports for the first time *C. paolae* and *C. subfagineus* in the country. The revised checklist includes 54 species of *Culicoides* recorded from the territory of Morocco. However, it is noted that the southernmost Saharan region of Eddakhla-Oued Eddahab has, to our knowledge, never been prospected (Fig. 1). The neighboring region of Laayoune-Sakia El Hamra has only been prospected around Laayoune in 2009–2010 [29]. The few specimens collected belonged to *C. imicola*, and to Newsteadi and Circumscriptus groups of species. Therefore, we have not included these two regions in Table 2. The small number of *Culicoides* specimens collected in Laayoune may be related to the arid climate prevailing in this region.

**Table 2:** *Culicoides* species list for Morocco by region

Species	Tanger-Tetouan-Alhoceima	Oriental	Fès-Meknès	Rabat-Salé-Kénitra	Beni Mellal-Khénifra	Casablanca-Settat	Marrakesh-Safi	Drâa-Tafilalet	Souss-Massa	Guelmim-Oued Noun
*C. azerbajdzhanicus*			[4]		[4]		[3, 4]		[6]	[4, 6]
*C. badooshensis*	[3]		[4]	[4, 6]			[3, 6]			
*C. calloti*									[6, 7]	[6, 7]
*C. catanei*	[6]			[4, 6]						
*C. circumscriptus*	[3, 6]	[3]	[4, 6]	[1, 3, 4, 6, 9]		[4, 6]	[4, 6]		[6]	[4]
*C. clastrieri*			^a^							
*C. derisor*			[4]	[4, 6]						
*C. duddingstoni*			^a^							
*C. dzhafarovi*				[4]						[6]
*C. faghihi*									[5]	
*C. fagineus*				[4, 6]	[4]		[4]			
*C. festivipennis*	[1, 3, 6]			[4, 6, 9]		[6]	[3, 6]			
*C. heteroclitus*							[5]		[5]	
*C. imicola*	[8]	[8]	[4, 8]	[8, 9]	[8]		[3, 8]	[8]	[8]	[4, 6, 8]
*C. jumineri*		[6]	[4]	[4, 6, 9]			[6]		[6]	[4, 6]
*C. kibunensis*			[5]				[5]			
*C. kingi*									[2]	
*C. kurensis*				[9]						
*C. landauae*			[5]							
*C. langeroni*				[4]			[3]			[6]
*C. longipennis*			[4]				[4]			
*C. marcleti*			[4]							[4]
*C. maritimus*	[4]			[3, 4]			[6]			
*C. montanus*	[6]			[4]			[4, 6]			[4]
*C. newsteadi*	[1, 3, 6]		[4]	[3, 4, 6, 9]	[4, 6]	[4, 6]	[3, 6]	[3]	[3]	[4]
*C. obsoletus*	[6, 8]	[8]	[8]	[1, 3, 4, 6, 8]	[4, 8]	[6, 8]	[4, 6, 8]		[8]	[8]
*C. odiatus*			[4]	[4, 6, 9]			[3, 6]		[5]	[4, 6]
*C. pallidicornis*										[4]
*C. pallidus*										[4]
*C. parroti*				[4]			[3, 6]			
*C. pictipennis*				[3]						
*C. picturatus*	[3]			[4]	[6]	[6]				
*C. pseudopallidus*			[4]				[3, 4]			
*C. pulicaris*	[8]	[8]	[4, 8]	[4, 6, 8]	[4, 8]	[8]	[6, 8]	[8]	[8]	[4, 6]
*C. pumilus*			[5]				[5]			
*C. punctatus*	[1, 3]		[4]	[3, 4]		[6]				
*C. puncticollis*	[3]		[4, 6]	[1, 3, 4, 6, 9]			[3, 6]			
*C. ravus*							[4]		[6]	[4, 6]
*C. saevus*	[3]	[6]	[4, 6]	[1, 4, 9]			[1, 3, 6]	[6]	[6]	[4, 6]
*C. sahariensis*	[3]	[3]	[4]	[3, 4, 6, 9]			[3]	[3]		[4, 6]
*C. santonicus*	[3]			[4, 6]		[6]				
*C. scoticus*					[4]	[6]	[4, 6]			
*C. sejfadinei*		[6]						[6]		[4, 6]
*C. semimaculatus*	[5]					[5]				
*C. sergenti*		[6]								[6]
*C. shaklawensis*			[5]				[5]			
*C. schultzei*							[1]			
*C. similis*										[4]
*C. simulator*			[5]							
*C. subfasciipennis*	[6]		[4]	[4, 9]	[4]					
*C. truncorum*				[4]	[4]					
*C. univittatus*	[4, 6]		[6]	[3, 4, 6]			[6]			
*C. vidourlensis*	[3]			[3]			[3]			

*Notes*: [1] Callot et al. (1968); [2] Bailly-Choumara & Kremer (1970); [3] Kremer et al. (1970); [4] Kremer et al. (1971); [5] Kremer et al. (1975); [6] Chaker et al. (1979);[7] Kremer et al. (1979); [8] Bouayoune et al. (1998); [9] Cêtre-Sossah & Baldet (2004)

^a^Unpublished records of Delécolle

Our review of the literature highlighted the use of synonym names for 11 species (*C. sahariensis*, *C. imicola*, *Culicoides univittatus* Vimmer, 1932, *C. newsteadi*, *Culicoides odiatus* Austen, 1921, *C. festivipennis*, *Culicoides pallidus* Khalaf, 1957, *Culicoides ravus* de Meillon, 1936, *Culicoides kibunensis* Tokunaga, 1937, *Culicoides sergenti* Kieffer, 1921 and *Culicoides truncorum* Edwards, 1939). Some authors also re-evaluated their own collections. *Culicoides riethi* Kieffer, 1914 erroneously reported [17], was modified to *C. puncticollis* in 1971 [18]. Caution should therefore be exercised when using former publications. It is mandatory to refer to the world catalogue for *Culicoides* [38] to validate species names and update them if needed. Moreover, our literature review highlighted the lack of identification tools for *Culicoides* in Morocco, and more broadly in North Africa, as already published for the Western Palaearctic region [33].

The updated checklist for the country was established after a critical analysis of the literature and from our novel collections. *Culicoides begueti* Clastrier, 1957 is mentioned by Kremer et al. [18] from unpublished collections in the Marrakech region. As materials and references to these catches were not available, the species has been removed from the list pending further reports. The presence of individuals belonging to the ‘Kingi-schultzei group’ was reported by Bailly-Choumara & Kremer in Souss-Massa Region [17, 21]. According to our molecular and morphological analysis, this species could be *C. kingi*. *Culicoides helveticus* was collected as a unique individual by Lhor et al. [29]. This species can be confused with *Culicoides stigma* (Meigen, 1818) and *Culicoides parroti* Kieffer, 1922, which have the same wing pattern, but can be distinguished by the shape of the spermatheca [40]. In our collections, *C. parroti* was observed. Considering the known distribution of *C. helveticus* is the mountainous regions of the Palaearctic region such as in Russia [41–43], France [40], Sweden [44] and Ukraine [45], we considered it was appropriate to remove this species from Moroccan list of *Culicoides*.

Our molecular analysis revealed that the specimens morphologically identified as *C. obsoletus*/*C. scoticus* belong to *C. obsoletus*, *C. scoticus* and one specimen of *C. montanus*. Indeed, *C. montanus* is a rare species that is phylogenetically very close to *C. obsoletus* [46, 47] for which the taxonomic status is a little controversial [48, 49]. Recently, several authors have reported the existence of cryptic diversity within the *C. obsoletus* taxon [50, 51], namely *C. obsoletus* clade O2, *C. obsoletus* dark and one unnamed entity. Albeit from a limited number of individuals, we did not observe such cryptic diversity within the *C. obsoletus* taxon in Morocco.

A clade of 6 sequences (i.e. MK732297, MK732292, MK732298, MK732283, MK732284 and MK732286) received high bootstrap support. This clade is part of the same phylogroup as *C. santonicus* Callot, Kremer, Rault & Bach, 1966 which belongs to the Vexans group, grouping three species with *Culicoides brunnicans* Edwards, 1939 and *Culicoides vexans* (Staeger, 1839). We hypothesized that it is either a novel species or simply one without voucher sequences in molecular libraries. This could be investigated by increasing the number of voucher sequences to other Afrotropical or Palaearctic species. Moreover, the *C. jumineri* clade showed genetic variability that may indicate that cryptic species diversity is present within this clade.

To our knowledge, our study records *C. paolae* for the first time in Morocco, whereas it was previously known from Italy [52], Greece [53], Algeria [54] and Tunisia [55–57]. This species, largely distributed in the Mediterranean region, was not considered, until recently, to be associated with BTV transmission. Indeed, BTV genome was recovered from the thorax of field-collected individuals in Sardinia during BTV outbreaks [15], raising a possible role of this species in BTV transmission. Updating the distribution of this species is thus of primary interest. To our knowledge, we also describe *C. subfagineus* for the first time in the country. Our specimens were first morphologically identified as *Culicoides grisescens* Edwards, 1939 but tree reconstruction clearly assigned all three specimens to *C. subfagineus*. *Culicoides subfagineus* was recently reported for the first time in Portugal [58] and Germany [59]. Some authors confuse this species with *Culicoides fagineus* Edwards, 1939 and group it into *C. subfagineus/C. fagineus* as pairs of species [60].

Finally, Moroccan fauna is composed of species with a different geographical distribution and can be classified into three groups: (i) species with a wide distribution covering the Palaearctic region (from Scandinavia to the Mediterranean) such as *C. circumscriptus*, *C. clastrieri*, *C. duddingstoni*, *Culicoides dzhafarovi* Remm, 1967, *C. fagineus*, *C. festivipennis*, *C. jumineri*, *C. kibunensis*, *Culicoides kurensis* Dzhafarov, 1960, *Culicoides maritimus* Kieffer, 1924, *C. montanus*, *C. newsteadi*, *C. obsoletus*, *C. odiatus*, *C. parroti*, *Culicoides pictipennis* (Staeger, 1839), *Culicoides picturatus* Kremer & Deduit, 1961, *C. pulicaris*, *C. punctatus*, *C. puncticollis*, *Culicoides saevus* Kieffer, 1922, *C. scoticus*, *Culicoides shaklawensis* Khalaf, Ingram & Macfie, 1957, *Culicoides simulator* Edwards, 1939, *Culicoides subfasciipennis* Kieffer, 1919 and *Culicoides vidourlensis* Callot, Kremer, Molet & Bach, 1968; (ii) species distributed around the Mediterranean basin such as *Culicoides azerbajdzhanicus* Dzhafarov, 1962, *C. cataneii*, *Culicoides derisor* Callot & Kremer, 1965, *Culicoides faghihi* Navai, 1971, *Culicoides heteroclitus* Kremer & Callot, 1965, *C. jumineri*, *Culicoides langeroni* Kieffer, 1921, *Culicoides longipennis* Khalaf, 1957, *Culicoides marcleti* Callot, Kremer & Basset, 1968, *C. paolae*, *Culicoides pseudopallidus* Khalaf, 1961, *C. sahariensis*, *C. santonicus*, *Culicoides sejfadinei* Dzhafarov, 1958, *Culicoides semimaculatus* Clastrier, 1958, *Culicoides sergenti* Kieffer, 1921, *C. subfagineus*, *C. univittatus*; and (iii) Afrotropical species also present in the southern part of the Palaearctic zone, especially in the Mediterranean such as *C. imicola*, C. *kingi*, *C. ravus* and *Culicoides similis* Carter, 1920.

Some species have limited numbers of records in the literature making their known geographical distribution too sporadic to be assigned to the above groups: *Culicoides badooshensis* Khalaf, 1961 (reported in Irak, Iran, Morocco and Turkey), *C. pallidus* (Morocco), *C. landauae* (Morocco), *C. calloti* (Morocco) and *Culicoides pumilus* (Winnertz, 1852) (Ukraine, France and Morocco).

The two other countries of the Maghreb, Algeria and Tunisia, have many bio-climatic similarities such as a Mediterranean coastline in the north and Sahara desert in the south. The *Culicoides* fauna of these countries has been studied in two steps, as in Morocco. First, inventories were carried out in the 1950s and the 1980s in Algeria [61, 62], leading to the record of 31 species, and in the 1980s in Tunisia [63], leading to the record of 22 species. Then, inventories were completed during studies following the BTV emergence in the 2000s in the Maghreb and in the whole Mediterranean region. Today, the number of species recorded in Morocco (54 species) was slightly higher than in Algeria with 47 species [54] and in Tunisia with 35 species [55, 57, 64, 65]. The same increasing species richness gradient from Tunisia to Morocco is observed for mosquitoes [66], and may be related to the greater diversity of climates and landscapes found in Morocco, including the influence of the ocean climate prevailing on the northwest coast and not existing in the two neighboring countries.

## Conclusions

The *Culicoides* fauna of Morocco includes 54 species, at least 8 of which are recognized vectors or potential vectors of *Culicoides*-borne viruses of economic importance in animal production. Among these, *C. imicola* is a proven BTV and AHSV vector [6, 7, 67] and was considered for a long time the only important vector. Most epidemiological studies have therefore focused on this species [23]. In this study, we confirmed by molecular assay the presence of both *C. obsoletus* and *C. scoticus* in Morocco, which are considered as probable BTV vectors in the western Palaearctic region [68, 69]. Moreover, we reported the presence of *C. kingi*, a potential EHDV vector [70, 71] and of *C. paolae*, which, together with *C. newsteadi*, *C. pulicaris* and *C. punctatus* [14, 15, 72], is regarded as a potential BTV vector. Establishing a comprehensive checklist of *Culicoides* for Morocco is a prerequisite for developing a barcode library and atlas of diagnostic characters, but also to plan further ecological studies, including large scale *Culicoides* collections focusing species of veterinary interest, to be able to carry out risk mapping for related *Culicoides-*borne diseases.

## Data Availability

Specimens are stored in ethanol with one specimen per species slide-mounted and are available upon request to Maria Bourquia and Khalid Khallaayoune at Agronomic and Veterinary Institute Hassan II, Rabat, Morocco.

## Abbreviations

AHSV: African horse sickness virus
BTV: bluetongue virus
EHDV: epizootic hemorrhagic disease virus
